# Dissemination of multiple carbapenem-resistant clones of *Acinetobacter baumannii* in the Eastern District of Saudi Arabia

**DOI:** 10.3389/fmicb.2015.00634

**Published:** 2015-07-02

**Authors:** Abdulrahman A. Al-Sultan, Benjamin A. Evans, Elsayed Aboulmagd, Ahmed A. Al-Qahtani, Marie Fe F. Bohol, Mohammed N. Al-Ahdal, Andres F. Opazo, Sebastian G. B. Amyes

**Affiliations:** ^1^College of Medicine, King Faisal University, Al Ahsa, Saudi Arabia; ^2^Department of Biomedical and Forensic Sciences, Faculty of Science and Technology, Anglia Ruskin University, Cambridge, UK; ^3^Department of Infection and Immunity, Research Center, King Faisal Specialist Hospital and Research Center, Riyadh, Saudi Arabia; ^4^Department of Microbiology and Immunology, Alfaisal University College of Medicine, Riyadh, Saudi Arabia; ^5^Liver Disease Research Center, King Saud University, Riyadh, Saudi Arabia; ^6^Department of Pathology and Laboratory Medicine, King Faisal Specialist Hospital and Research Center, Riyadh, Saudi Arabia; ^7^Laboratorio de Investigación en Agentes Antibacterianos, Facultad de Ciencias Biológicas, Universidad de Concepción, Concepción, Chile; ^8^Medical Microbiology, University of Edinburgh, Edinburgh, UK

**Keywords:** *Acinetobacter baumannii*, carbapenem resistance, Saudi Arabia, PFGE, OXA, VIM

## Abstract

It has previously been shown that carbapenem-resistant *Acinetobacter baumannii* are frequently detected in Saudi Arabia. The present study aimed to identify the epidemiology and distribution of antibiotic resistance determinants in these bacteria. A total of 83 *A. baumannii* isolates were typed by pulsed-field gel electrophoresis (PFGE), and screened by PCR for carbapenemase genes and insertion sequences. Antibiotic sensitivity to imipenem, meropenem, tigecycline, and colistin were determined. Eight different PFGE groups were identified, and were spread across multiple hospitals. Many of the PFGE groups contained isolates belonging to World-wide clone 2. Carbapenem resistance or intermediate resistance was detected in 69% of isolates. The *bla*_VIM_ gene was detected in 94% of isolates, while *bla*_OXA–23–like_ genes were detected in 58%. The data demonstrate the co-existence and wide distribution of a number of clones of carbapenem-resistant *A. baumannii* carrying multiple carbapenem-resistance determinants within hospitals in the Eastern Region of Saudi Arabia.

## Introduction

A number of recent studies have identified high levels of carbapenem resistance in isolates of *Acinetobacter baumannii* from Saudi Arabia (Alsultan et al., 2013; Abdalhamid et al., 2014; Aly et al., 2014). These studies have demonstrated that a large number of different genetic lineages within a hospital are resistant to the carbapenems, rather than there being the predominance of a single epidemic clone (Abdalhamid et al., 2014; Aly et al., 2014). These isolates often belong to widespread clonal lineages (Zarrilli et al., 2013). However, due to examining only isolates obtained from a single hospital (Abdalhamid et al., 2014; Aly et al., 2014), or the absence of molecular typing data (Alsultan et al., 2013), it is still not clear what the epidemiological pattern of carbapenem-resistant *A. baumannii* is on a wider scale within Saudi Arabia.

It was previously shown in a study utilizing relatively few isolates that a number of different lineages of *A. baumannii*, defined by pulsed-field gel electrophoresis (PFGE), were associated with infections in diabetic patients in Saudi Arabia, with one of these lineages found in more than one hospital (Alsultan et al., 2009). A much larger study found that isolates from diabetic patients were more likely to be carbapenem resistant and carry an acquired OXA-type β-lactamase, but did not identify the molecular epidemiology of the isolates (Alsultan et al., 2013). A recent study carried out in Abu Dhabi, United Arab Emirates, that borders Saudi Arabia, identified a diverse range of isolates, with even the epidemic isolates belonging to multiple PFGE pulsotypes and genotypes (Sonnevend et al., 2013).

The high and increasing prevalence of type 2 diabetes is a very serious health concern in Saudi Arabia, and as these patients are particularly susceptible to bacterial infection, the prevalence of carbapenem-resistant *A. baumannii* in this group is of particular concern (Prata-Rocha et al., 2012). Here we have typed a larger collection of 83 *A. baumannii* isolates taken from a combination of diabetic and non-diabetic patients from the Eastern District of Saudi Arabia. We aim to determine the epidemiological relationships between isolates and the distribution of antibiotic resistance determinants in this bacterial population, in order to address the lack of molecular epidemiological data between hospitals in a wider geographic context.

## Materials and Methods

A total of 83 isolates were collected between 2008 and 2012 from different specimens from patients from 7 major hospitals in the Eastern province of Saudi Arabia—23 isolates from patients with type 2 Diabetes Mellitus, and 60 isolates from patients without this condition. For the purpose of our study, patients were considered to be diabetic if they were using insulin and were undergoing hospital treatment for the condition. All isolates were identified presumptively by the Vitek compact II system as being *A. baumannii*.

Multiplex polymerase chain reactions (PCRs) using primers for the *bla*_OXA–51–like_, *bla*_OXA–23–like_, *bla*_OXA–40–like_, and *bla*_OXA–58–like_ genes were performed (Woodford et al., 2006), with species identification being confirmed by the production of a *bla*_OXA–51–like_ amplicon. Amplification and sequencing of complete *bla*_OXA–51–like_ alleles was performed using the primers OXA-69A and OXA-69B (Heritier et al., 2005), and the allele identity used to determine the epidemic lineage to which the isolate belonged as first described by Evans et al. (2008) and later confirmed by Pournaras et al. (2014). Isolates were screened by PCR for the presence of acquired metallo-β-lactamase genes (MBLs) of the *bla*_IMP_, *bla*_VIM_, *bla*_NDM_, *bla*_SIM_, and *bla*_GIM_ (Ellington et al., 2007; Poirel et al., 2011) families using consensus primers, and for the insertion sequences IS*Aba1*, IS*Aba2*, IS*Aba3*, and IS*18* (Poirel and Nordmann, 2006).

The minimum inhibitory concentrations (MICs) of imipenem, meropenem, tigecycline and colistin were determined by the agar plate doubling-dilution method according to the British Society for Antimicrobial Chemotherapy (BSAC) guidelines (Andrews, 2010).

Isolates were typed by PFGE using the method described by Miranda et al. (1996) as previously described (Evans et al., 2008). PFGE profiles were analyzed using the Bionumerics software version 6.5. All data were analyzed in SPSS version 20 (SPSS Inc.,). Following Kruskal Wallis tests, differences between groups were determined by pairwise Mann-Whitney U tests. Correction for multiple testing was performed using the method of Benjamini and Hochberg (1995).

## Results

**FIGURE 1:**
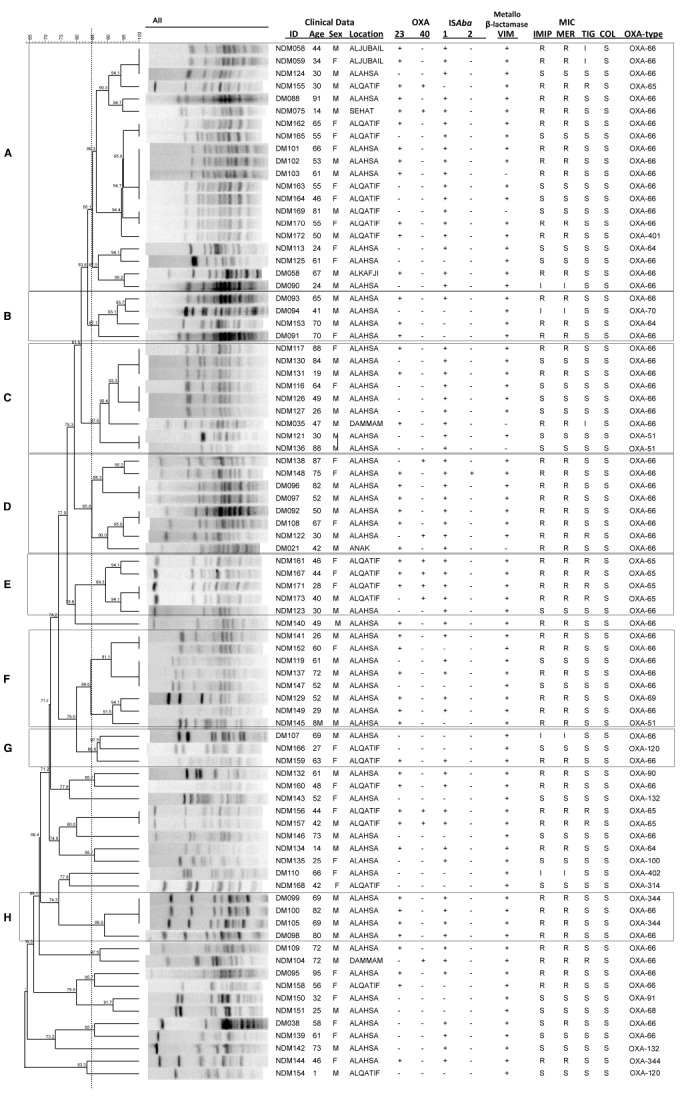
**Isolate relatedness, antibiotic resistances and molecular screening of isolates.** In the ID column, DM stands for Diabetes Mellitus, NDM for non-Diabetes Mellitus. IMIP, imipenem; MER, meropenem; TIG, tigecycline; COL, colistin. **(A–H)** Indicate PFGE groups assigned as described in the text.

Isolates were grouped according to their PFGE profiles (Figure 1). Sixty-two isolates were assigned to one of eight different PFGE groups, defined as having PFGE profiles with 85% similarity or greater, and consisting of at least three isolates. The remaining 21 isolates that did not meet these criteria were considered to be ungrouped. There was a significant difference in the diabetic status of the patients between the individual PFGE groups (Kruskal Wallis, χ^2^ = 26.32, d.f. = 7, *p* = 0.0024 following correction), with isolates from diabetic patients being in the majority in PFGE groups B, D, and H (Figure 1).

**FIGURE 2:**
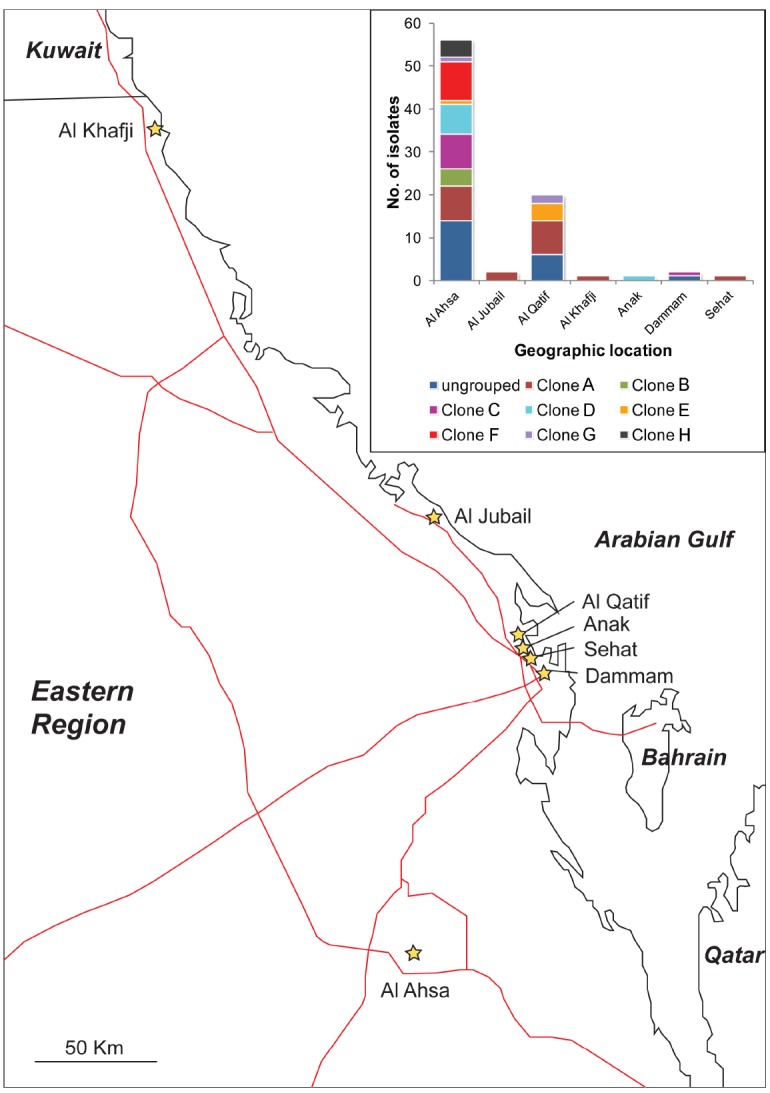
**Location of hospitals from where isolates were obtained, and (insert) the distribution of PFGE clones (as defined in Figure 1) by their geographic location.** Hospitals are shown as stars, and major roads as red lines.

The majority of the isolates (67%) were from Al Ahsa, with most of the remaining isolates (24%) originating from Al Qatif. In Al Ahsa, isolates representing all eight of the PFGE groups were identified (Figure 2). Three of these groups were also identified in Al Qatif. Additionally, isolates from one or more of the PFGE groups were identified at the five other locations sampled, despite the geographic distance between these hospitals (Figure 2).

Of the 83 isolates, 57 (69%) were resistant or had intermediate resistance to at least one carbapenem. In agreement with previous findings (Alsultan et al., 2013), isolates from diabetic patients were more likely to be resistant to a carbapenem than those from non-diabetic patients (diabetic status *vs* meropenem resistance, Mann Whitney U test, *Z* = –3.787, *p* = 0.001 following correction). A total of 13 isolates (16%) were resistant to tigecycline, and none to colistin. A large proportion of isolates were found to carry resistance genes, with 48 isolates (58%) carrying *bla*_OXA–23–like_ genes, 11 isolates (13%) carrying *bla*_OXA–40–like_ genes, and 78 isolates (94%) carrying a *bla*_VIM_ gene. PCRs for *bla*_OXA–58–like_, *bla*_IMP_, *bla*_NDM_, *bla*_SIM_, and *bla*_GIM_ genes were all negative. There was a significant difference in the presence of *bla*_OXA–40_ genes between the different PFGE groups (Kruskal Wallis, χ^2^ = 25.12, d.f. = 7, *p* = 0.003 following correction), with the gene being detected with particularly high frequency in PFGE group E (Figure 1). Phenotypic resistance to the carbapenems was associated with the presence of a *bla*_OXA–23–like_ gene rather than the presence of a *bla*_VIM–like_ gene (meropenem resistance *vs bla*_OXA–23–like_ carriage: Mann Whitney U test, *Z* = –7.162, *p* < 0.00001 following correction; meropenem resistance *vs bla*_VIM_ carriage: Mann Whitney U test, *Z* = –0.429, *p* = 0.713 following correction).

A total of 65 isolates (78%) were positive by PCR for IS*Aba1*, while only two isolates were positive for IS*Aba2*. The insertion sequences IS*Aba3* and IS*18* were not detected. The different PFGE groups differed significantly in the presence of IS*Aba1* (Kruskal Wallis, χ^2^ = 23.06, d.f. = 7, *p* = 0.004 following correction) with higher frequencies of detection of this element in PFGE groups A, C, D, E, and H (Figure 1).

**FIGURE 3:**
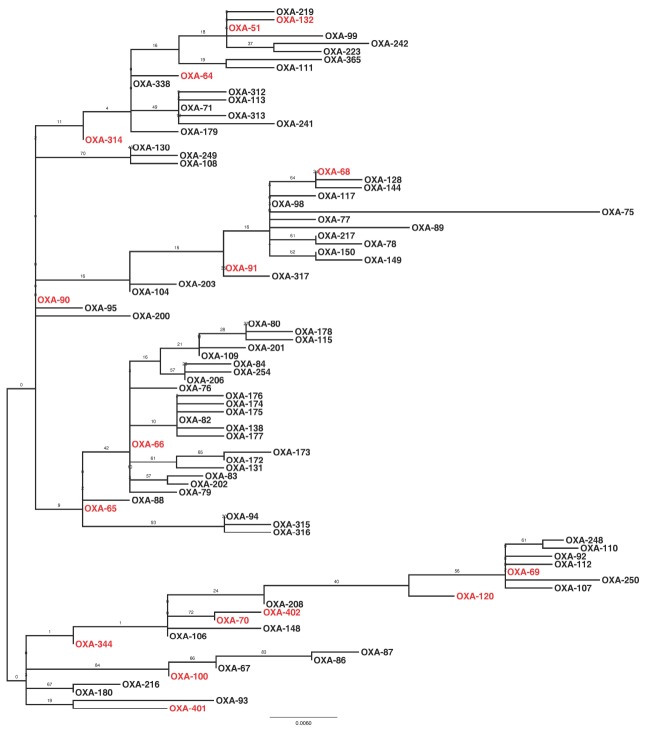
**Maximum likelihood phylogeny based upon amino-acid sequences of OXA-51-like enzymes.** Enzymes that were encoded by isolates in the present study are highlighted in red. The phylogeny is mid-point rooted, with support for the branches estimated from 100 bootstraps.

Sequencing of the *bla*_OXA–51–like_ alleles identified the presence of the *bla*_OXA–66_ allele in 54 isolates (65%), with the remaining 29 isolates encoding one of 15 other OXA-51-like enzymes (Figures 1 and 3). Isolates carrying a *bla*_OXA–66_ allele were considered to belong to the World-wide clone 2 (WW2) lineage (Evans et al., 2008; Higgins et al., 2009). Isolates belonging to one of the PFGE groups were more likely to encode a *bla*_OXA–66_ allele, while isolates that were not grouped were more likely to encode a non-*bla*_OXA–66_ allele (Mann Whitney U test, *Z* = –2.981, *p* = 0.012 following correction). In addition there was a significant difference in the association of IS*Aba1* with *bla*_OXA–51–like_ alleles, with the insertion sequence found significantly more often upstream of *bla*_OXA–66_ alleles than non-*bla*_OXA–66_ alleles (Mann Whitney U test, *Z* = –3.171, *p* = 0.011 following correction).

## Discussion

Carbapenem-resistant *A. baumannii* pose a significant challenge to the treatment of infected patients. These patients are usually immunocompromised, and the elimination of the carbapenem antibiotics as an effective therapy leaves very few treatment options open. In the current study, *A. baumannii* isolates from hospitals in the Eastern District of Saudi Arabia were examined to identify their mechanisms of resistance to the carbapenems and their local epidemiology, both within the hospitals and the District.

Typically, the isolation of many carbapenem-resistant bacteria within a hospital may be explained by two epidemiological patterns: the emergence and spread of a particularly successful clone, or the persistence and co-existence of many clonal lineages, potentially with importation from the community. Our data are most consistent with the latter scenario for two reasons. Firstly, many different PFGE groups were identified both within Al Ahsa and Al Qatif. Moreover, isolates from the same PFGE groups were identified in different hospitals. It is also noteworthy that 25% of the isolates were not assigned to a PFGE group. The distribution of isolates from the same PFGE group across the Eastern District (Al Kafji and Al Ahsa are ∼430 km apart by road, Figure 2) and the identification of many ungrouped carbapenem-resistant isolates suggest the persistent co-existence of many lineages across the region. Secondly, there is a large degree of variation in antibiotic resistance phenotypes and genotypes within PFGE groups. The only features that were significantly associated with PFGE groups were the presence of *bla*_OXA–40–like_ genes, and the presence of IS*Aba1*. However, these genes were still identified in isolates belonging to PFGE groups that were not significantly associated with their carriage. Antibiotic resistance phenotypes and other genes associated with resistance were distributed amongst PFGE groups and ungrouped isolates, with no statistically significant positive or negative association with any PFGE group. This suggests that these genes are endemic within the general *A. baumannii* population where they have either spread amongst the many genetic lineages, or they have existed within the *A. baumannii* population for a substantial period of time as the different genetic lineages have diverged, with some genes being lost over time. Taken together, these data are consistent with the endemicity of a polyclonal carbapenem-resistant or carbapenemase-encoding population of *A. baumannii* in the Eastern District of Saudi Arabia.

Previous studies have identified carbapenem-resistant *A. baumannii* isolates belonging to the WW2 lineage as being widespread and responsible for outbreaks of infection in hospitals globally (Higgins et al., 2009; Karah et al., 2012). In the current study, the majority of isolates were considered to belong to WW2 by virtue of their specific *bla*_OXA–51–like_ allele (Evans et al., 2008; Higgins et al., 2009). A previous study associated isolates carrying *bla*_OXA–64_ with multi-locus sequence typing (MLST) clonal complex (CC) 25, *bla*_OXA–51_ with CC15, *bla*_OXA–100_ with CC32, and *bla*_OXA–65_ with CC79 from the Pasteur MLST scheme (Pournaras et al., 2014). As such, isolates identified in the present study may also belong to these clonal complexes. Despite the fact that many different PFGE groups were identified, isolates belonging to one of these PFGE groups were statistically more likely to belong to WW2 than the ungrouped isolates. In addition, these WW2 isolates were statistically more likely to encode an IS*Aba1* element upstream of the *bla*_OXA–51–like_ genes, which has been implicated in increased expression of the β-lactamase gene (Turton et al., 2006). As has been identified previously (Evans et al., 2008; Hamouda et al., 2010), PFGE profiles did not correlate perfectly with the *bla*_OXA–51–like_ gene content of the isolates in a few cases, which may be explained by potential mobilization of the *bla*_OXA–51–like_ genes (Chen et al., 2010). Together these data are consistent with previous studies, and demonstrate that the different PFGE groups identified in this study are subgroups of WW2, and therefore this global clone represents the majority of carbapenem-resistant isolates in the Eastern District.

The question arises as to the precise nature of the local epidemiology of carbapenem resistance in these isolates. There are two scenarios that can be explored. Firstly, a series of endemic clones may be circulating within and amongst hospitals in the Eastern District, with the transmission of plasmids carrying antibiotic resistance determinants responsible for a large degree of the variation in antibiotic resistance phenotype and genotype between strains. Secondly, carbapenem-resistant *A. baumannii* may be endemic within the community, with strains being imported into the hospital where they briefly spread, before being replaced by other strains. Most of the isolates (94%) in the current study were collected between October 2010 and April 2011, and as such an assessment cannot be made from these data of the changes in frequency of different strains over time. Establishing which of these scenarios reflect the situation in the Eastern District will enable the appropriate infection control advice to be given to hospitals to reduce the prevalence of carbapenem-resistant *A. baumannii*, and is the subject of our future research.

## Author Contributions

AAS, SA conceived and designed the study. AAS, EA, AAQ, MB, MA, AO acquired the data. AAS, BE, EA analyzed the data. AAS, BE, EA, SA drafted and critically evaluated the manuscript. All authors approved the final version of the manuscript.

### Conflict of Interest Statement

The authors declare that the research was conducted in the absence of any commercial or financial relationships that could be construed as a potential conflict of interest.
